# Olfactory consciousness across disciplines

**DOI:** 10.3389/fpsyg.2014.00931

**Published:** 2014-08-22

**Authors:** Andreas Keller, Benjamin D. Young

**Affiliations:** ^1^Philosophy Program, Graduate Center, City University of New YorkNew York, NY, USA; ^2^The Department of Cognitive and Brain Science, Ben-Gurion University of the NegevBeer-Sheva, Israel

**Keywords:** olfaction, smell, consciousness, theories of consciousness, perceptual consciousness

Although vision is the *de facto* model system of consciousness research, studying olfactory consciousness has its own advantages, as this collection of articles emphatically demonstrates. One advantage of olfaction is its computational and phenomenological simplicity, which facilitates the identification of basic principles. Other researchers study olfactory consciousness not because of its simplicity, but because of its unique features. Together, olfaction's simplicity and its distinctiveness make it an ideal system for testing theories of consciousness. In this research topic, the results of recent research into olfactory consciousness are presented.

## Simplicity of olfaction

The relative simplicity of olfaction makes it a natural starting point for investigating perception. Olfaction has been used as a theoretical launchpad at least since 1764, when Reid wrote that “beginning with the simplest, and proceeding by very cautious steps to the most complex” (Reid, 1764) is the best strategy to understand the human mind. Following his own advice, Reid begins his *Inquiry into the Human Mind* with a chapter on olfaction. Quilty-Dunn's article explores Reid's account of odors as secondary qualities and argues for the relevance of Reid's theory for contemporary debates (Quilty-Dunn, 2013). The simplicity of the olfactory system is also what prompts Merrick and her colleagues to suggest in their review article that olfaction can be used as “the gateway to the neural correlates of consciousness” (Merrick et al., 2014).

Keller, in his contribution, takes advantage of the relative simplicity of the olfactory system in his attempt to identify the function of conscious information processing. Visual perception performs a myriad of functions. This versatility of vision makes the identification of the subset of vision's functions that require consciousness challenging. This problem, Keller argues, is much more tractable in olfaction, which is a specialized sense with circumscribed functions (Keller, 2014).

## Unique features of olfaction

The many unique features that distinguish olfactory consciousness from other forms of perceptual consciousness are reviewed here with a focus on cognition by Stevenson and Attuquayefio (2013), and with a focus on neuroanatomy and neurodynamics by Merrick et al. (2014).

One striking feature of olfaction is the difficulty associated with olfactory imagery. It is easier to imagine seeing something than it is to imagine smelling it. Whether it is at all possible to voluntarily experiencing smells in the absence of the physical stimulus is the topic of ongoing research. Stevenson and Attuquayefio review the relevant literature and conclude that olfactory imagery is not possible (Stevenson and Attuquayefio, 2013). In contrast, Arshamian and Larsson argue that some individuals are capable of imagining smells (Arshamian and Larsson, 2014). Young (2014) sides with Arshamian and Larsson. According to Young, the fact that experiences during olfactory imagery have a qualitative character shows that olfactory awareness is always qualitatively conscious. In addition, he surveys evidence that olfactory sensory states can have a qualitative character in the absence of awareness.

Another peculiarity of olfaction is that it is unclear what, if anything, smells represent. Lycan, in his article, argues that olfaction does represent (Lycan, 2014). Lycan elaborates his previous proposal that a smell represents a miasma in the air (Lycan, 1996, 2000). In contrast, Batty, in her contribution, defends her proposal that there are no represented objects in olfaction (Batty, 2014). Instead, according to Batty, smells represent olfactory properties as occurring abstractly in our vicinity. Batty argues that, because there are no objects in olfaction, there can also be no object-failure, and therefore no olfactory illusions. A third view on the topic of olfactory objects is presented by Castro and Seeley (2014), who argue that the objects of olfactory perception are not objective physical entities, but affective categories.

The unique temporal structure of olfactory perception serves as the focus of Olofsson's review (Olofsson, 2014). Olfaction has a much lower temporal resolution than vision and audition. Olofsson reviews the literature on measurements of the time it takes subjects to respond to olfactory stimuli and proposes a cascade model of olfactory perception according to which we first detect an odor, then identify it, and finally determine odor valence and edibility.

## Theory testing in olfaction

Some contributions to this research topic show that the simplicity of olfaction and its distinctiveness make it a useful system for testing theories of consciousness. Quality-space theory explains the nature of the mental qualities distinctive of perceptual states by appeal to their role in perceiving rather than by appeal to conscious subjective reports (Rosenthal, 2010). Here, Young and his coauthors show that quality-space theory, which is typically described in terms of color qualities, also applies to odor qualities (Young et al., 2014).

A second theory that is discussed here is the global workspace theory, according to which widespread broadcasting of information in the brain is necessary for consciousness (Baars, 2005). It has previously been suggested that the global workspace theory fails to explain conscious odor perception (Young, 2012). Here, Baars defends the global workspace theory against this suggestion and argues that the theory is applicable to olfactory consciousness (Baars, 2013). The widespread broadcasting of information that is central for the global workspace theory may be accomplished through synchronous gamma oscillations. Mori and colleagues review what is known about these oscillations in olfactory networks (Mori et al., 2013). They concluded that in olfaction the olfactory bulb plays the role that the thalamus plays for visual consciousness.

## Outlook

*Olfactory Consciousness across Disciplines* demonstrates that the simplicity and distinctiveness of olfactory consciousness make it an ideal system for theory testing. Theories of consciousness that aspire to be applicable to all modalities have to be consistent with these results. This research topic provides the resources for testing theories of consciousness in olfaction and offers some examples. We hope that this will enable and encourage a more systematic investigation of olfactory consciousness.

### Conflict of interest statement

The authors declare that the research was conducted in the absence of any commercial or financial relationships that could be construed as a potential conflict of interest.
